# Supplementary material for: Construction of Artificial Ovaries with Decellularized Porcine Scaffold and Its Elicited Immune Response after Xenotransplantation in Mice
Source: J Funct Biomater. 2022 Sep 28;13(4):165. doi: 10.3390/jfb13040165 (PMC9589985; doi:10.3390/jfb13040165)
Supplement: Supplementary file 1 [file jfb-13-00165-s001.zip › jfb-1892565-supplementary.pdf]

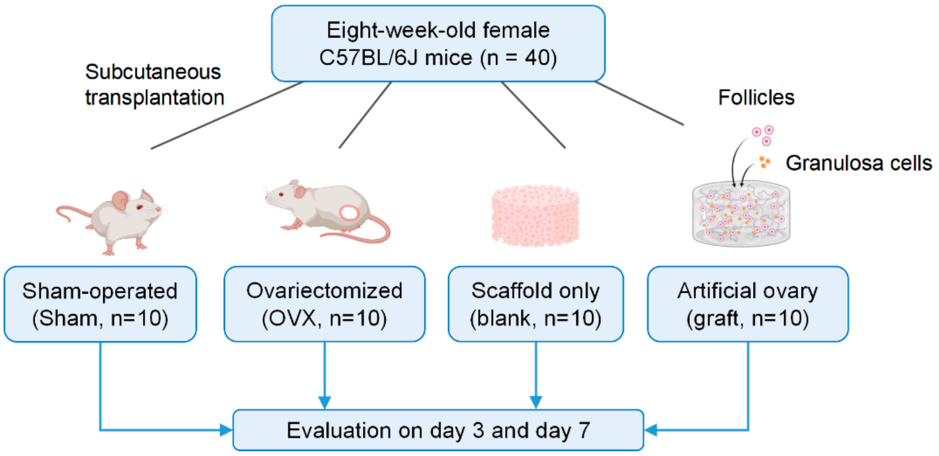

Figure S1. The flowchart of fabricating artificial ovaries using Dec scaffold and the design of *in vivo* experiments. Abbreviation: OVX, ovariectomized.

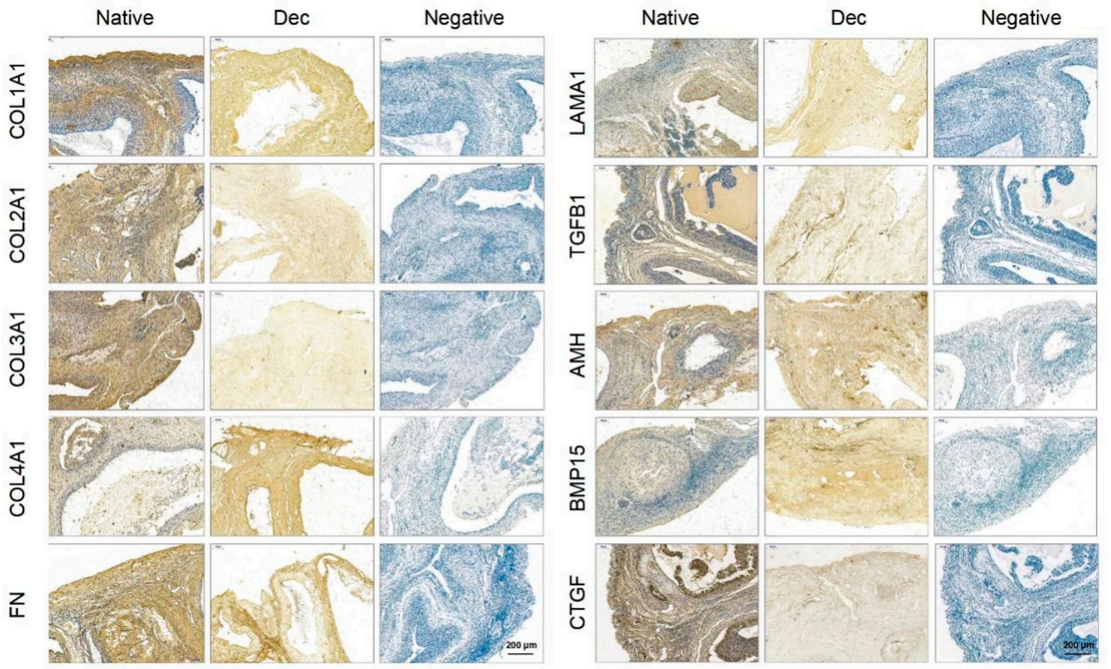

Figure S2. IHC staining showed the distribution of the ECM proteins in native and Dec tissues.

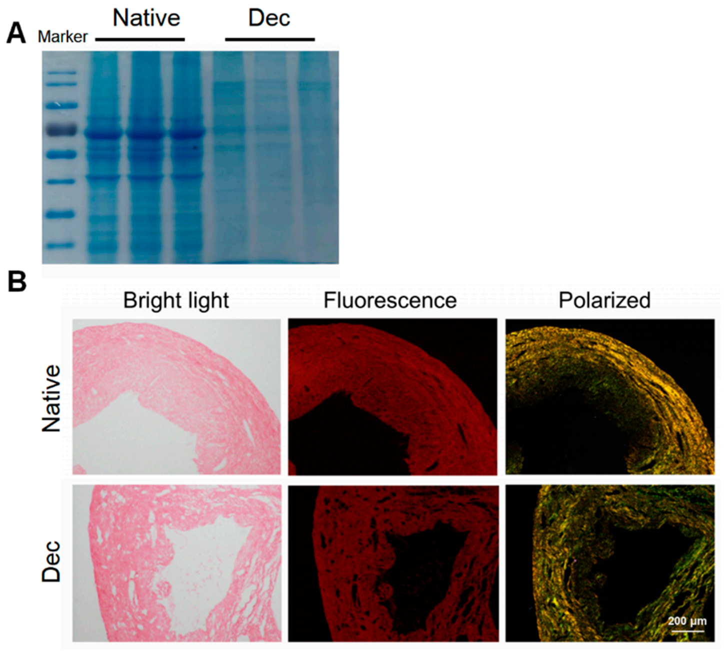

Figure S3. The identification of ECM proteins. (A) Coomassie blue G-250 staining demonstrated the reduction of proteins after decellularization (n = 3 each group). (B) Observation of collagen fibers stained by picrosirius red under the bright light, fluorescence and polarized light.

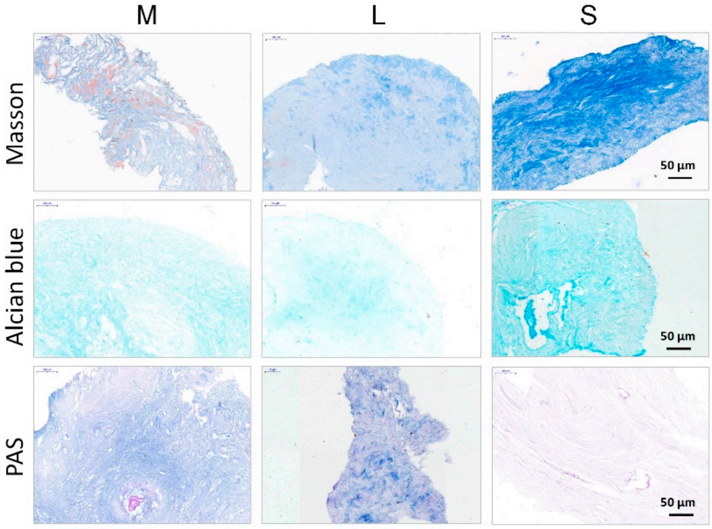

Figure S4. The connective tissue staining of Dec tissues reproduced according to proposed protocols.

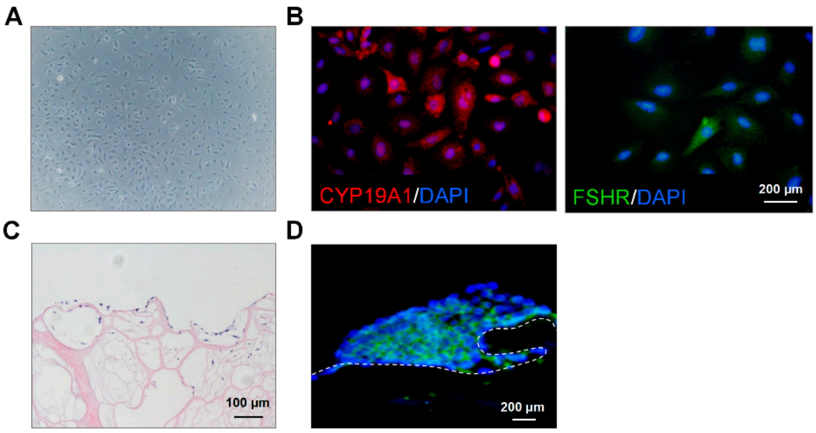

Figure S5. The isolation and culture of murine GCs. (A and B) Identification of the isolated mice ovarian cells. (C) The ovarian cells are seeded and some migrated into the decellularized scaffolds on day 7. (D) Representative image of ovarian cell clusters adhered to the scaffolds.

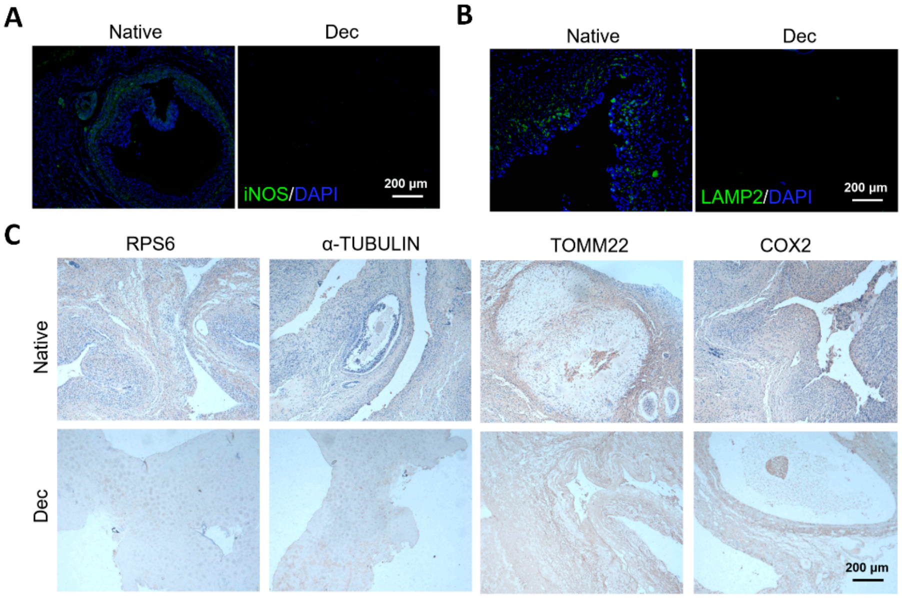

Figure S6. The identification of organelle residues after decellularization. Representative immunofluorescence images of iNOS (A) and LAMP2 (B) indicated the clearance of peroxisome and lysosome during decellularization. (C) Representative immunohistochemistry images of RPS6 and α-TUBULIN demonstrated the absence of ribosome and cytoskeleton after decellularization, while a small amount of mitochondrion and endosome residues could be observed. Abbreviation: iNOS, inducible nitric oxide synthase; LAMP, lysosomal associated membrane protein; RPS6, ribosomal protein S6; TOMM, translocase of outer mitochondrial membrane; COX, cyclooxygenase.

Supplementary table S1. Quantification comparisons between different ovarian decellularization protocols

Year	Study	DNA	Extracellular matrix	Species
2022	This study	12.86 ± 1.707 ng/mg	Collagen (ns, 7.624 ± 0.8 µg/mg)	Pig
2021	Alaee *et al.*[1]	21.45 ± 3.36 ng/mg	-	Rat
2017	Brown *et al.*[2]	175 ng/mg	-	Pig
2021	Chiti *et al.*[3]	0	Collagen (ns, 30.67 ± 0.2 mg/mg)	Bovine
2019	Eivazkhania *et al.*[4]	over 1000 ng/mg or lower than 500 ng/mg according to the species	Collagen, GAG (not given)	Mouse, sheep and human
2022	Haghshenas *et al.*[5]	114 ± 32.46 ng/mg	Collagen (ns), GAG (decrease, 166.2 ± 5.87)	Human
2018	Hassanpour *et al.*[6]	40 ± 7.33 ng/mg	-	Human
2017	Liu *et al.*[7]	20.92 ± 4.56 ng/mg	Collagen (ns, 321.8 ± 124.1 µg/mg), GAG (decrease, 85.1 ± 12.5 µg/mg)	Pig
2017	Motamed *et al.*[8]	39.38 ± 4.04 ng/mg	GAG (decrease, 43 ± 3.08 µg/mg)	Human
2021	Pennarossa *et al.*[9]	50 ± 30 ng/mg	-	Pig
2020	Pennarossa *et al.*[10]	30 ± 10 ng/mg	Collagen (ns, 49.9 ± 5.7 µg/mg), elastin (ns, 35.1 ± 1.7 µg/mg), GAG (ns, 5.2 ± 0.4 µg/mg)	Pig
2021	Sarabadani *et al.*[11]	8.98 ng/mg	-	Mouse
2021	Sistani *et al.*[12]	+ (not given)	-	Human

Abbreviations: GAG, glycosaminoglycan; ns, not significant (compared with the native tissues)

1. Alaee, S., et al., *The decellularized ovary as a potential scaffold for maturation of preantral ovarian follicles of prepubertal mice.* Syst Biol Reprod Med, 2021. **67**(6): p. 413-427.
2. Woodruff, M.M.L.L.R.N.S.K., *Artificial ovary*. 2017: United States.
3. Chiti, M.C., et al., *Ovarian extracellular matrix-based hydrogel for human ovarian follicle survival in vivo: A pilot work.* J Biomed Mater Res B Appl Biomater, 2022. **110**(5): p. 1012-1022.
4. Eivazkhani, F., et al., *Evaluating two ovarian decellularization methods in three species.* Mater Sci Eng C Mater Biol Appl, 2019. **102**: p. 670-682.
5. Haghshenas, M., et al., *Mouse ovarian follicle growth in an amniotic membrane-based hydrogel.*

J Biomater Appl, 2022: p. 8853282221094193.

6. Hassanpour, A., et al., *Decellularized human ovarian scaffold based on a sodium lauryl ester sulfate (SLES)-treated protocol, as a natural three-dimensional scaffold for construction of bioengineered ovaries.* Stem Cell Res Ther, 2018. **9**(1): p. 252.
7. Liu, W.Y., et al., *Xenogeneic Decellularized Scaffold: A Novel Platform for Ovary Regeneration.* Tissue Eng Part C Methods, 2017. **23**(2): p. 61-71.
8. Motamed, M., et al., *Tissue Engineered Human Amniotic Membrane Application in Mouse Ovarian Follicular Culture.* Ann Biomed Eng, 2017. **45**(7): p. 1664-1675.
9. Pennarossa, G., et al., *Ovarian Decellularized Bioscaffolds Provide an Optimal Microenvironment for Cell Growth and Differentiation In Vitro.* Cells, 2021. **10**(8).
10. Pennarossa, G., et al., *Whole-ovary decellularization generates an effective 3D bioscaffold for ovarian bioengineering.* J Assist Reprod Genet, 2020. **37**(6): p. 1329-1339.
11. Sarabadani, M., et al., *Co-culture with peritoneum mesothelial stem cells supports the in vitro growth of mouse ovarian follicles.* J Biomed Mater Res A, 2021. **109**(12): p. 2685-2694.
12. Sistani, M.N., et al., *Characteristics of a decellularized human ovarian tissue created by combined protocols and its interaction with human endometrial mesenchymal cells.* Prog Biomater, 2021. **10**(3): p. 195-206.
